# Ocular Symptoms in Adolescents and Young Adults With Electronic Cigarette, Cigarette, and Dual Use

**DOI:** 10.1001/jamaophthalmol.2023.3852

**Published:** 2023-08-31

**Authors:** Anne X. Nguyen, Shivani M. Gaiha, Sukyung Chung, Bonnie Halpern-Felsher, Albert Y. Wu

**Affiliations:** 1 Faculty of Medicine, McGill University, Montréal, Quebec, Canada; 2 REACH Lab, Division of Adolescent Medicine, Department of Pediatrics, Stanford University, Stanford, California; 3 Center for Population Health Sciences, School of Medicine, Stanford University, Stanford, California; 4 Department of Ophthalmology, Stanford University School of Medicine, Stanford, California

## Abstract

**Importance:**

Despite increasing use of cigarettes and electronic cigarettes (e-cigarettes) and related health effects among youth, few studies have reported their effects on eyes.

**Objective:**

To examine the frequency and severity of ocular symptoms (ocular discomfort, pain, burning, itching, redness, dryness, glare, blurriness, strain, and headaches) in young e-cigarette and cigarette users.

**Design, Setting, and Participants:**

In an observational cross-sectional study, a survey conducted in May 6 to 14, 2020, asked participants about use (ever, past 30 days, and past 7 days) of e-cigarettes and cigarettes. The participants included US individuals aged 13 to 24 years.

**Main Outcomes and Measures:**

Associations between vision-related outcomes (general vision, severity/frequency of ocular symptoms) and tobacco use were analyzed using weighted multivariable logistic regressions, adjusting for sociodemographic factors, contact lens use, and other combustible use.

**Results:**

There were 2168 never users, 2183 ever users, 1092 past 30-day users, and 919 past 7-day users of e-cigarettes; 55.9% of e-cigarette ever users also used cigarettes (dual users). Of the 4351 respondents, 63.8% identified as female, and mean (SD) age was 19.1 (2.9) years. Between 1.1% and 3.9% of ever dual users reported severe to very severe ocular symptoms; between 0.9% and 4.3% reported daily symptoms, which was higher than the proportion of symptoms in e-cigarette- or cigarette-only users. Past 7-day dual users had more severe itching (adjusted odds ratio [AOR], 2.37; 95% CI, 1.36-4.13; *P* = .002), redness (AOR, 2.58; 95% CI, 1.50-4.46; *P* = .001), dryness (AOR, 2.89; 95% CI, 1.64-5.08; *P* < .001), glare (AOR, 2.56; 95% CI, 1.50-4.35; *P* = .001), blurriness (AOR, 2.47; 95% CI, 1.36-4.50; *P* = .003), headaches (AOR, 2.31; 95% CI, 1.34-4.00; *P* = .003); and more frequent pain (AOR, 3.45; 95% CI, 2.09-5.68; *P* < .001), burning (AOR, 3.08; 95% CI, 1.86-5.09; *P* < .001), and redness (AOR, 2.72; 95% CI, 1.69-4.36; *P* < .001) than all other participants. Past 30-day dual users had more severe dryness (AOR, 2.65; 95% CI, 1.61-4.36; *P* < .001) and more frequent pain (AOR, 3.33; 95% CI, 2.12-5.21; *P* < .001) than all other participants. Ever dual users experienced more severe dryness (AOR, 1.60; 95% CI, 1.05-2.43; *P* = .03) and blurriness (AOR, 1.79; 95% CI, 1.21-2.64; *P* = .003) and more frequent pain (AOR, 1.69; 95% CI, 1.13-2.53; *P* = .01) and blurriness (AOR, 1.63; 95% CI, 1.13-2.36; *P* = .009) than never users.

**Conclusions and Relevance:**

In this cross-sectional US study, adolescents and young adult users of both e-cigarettes and cigarettes had a higher likelihood of experiencing severe and frequent ocular symptoms, with past 7-day users reporting more symptoms than past 30-day users or ever users. These findings provide additional reasons for users of e-cigarettes and cigarettes to reduce their tobacco use to possibly prevent or minimize ocular symptoms.

## Key Points

**Question:**

What is the frequency and severity of ocular symptoms reported by young users of electronic cigarettes (e-cigarettes) and cigarettes in the US?

**Findings:**

This cross-sectional study of 4351 individuals aged 13 to 24 years noted that users of both e-cigarettes and cigarettes have a higher likelihood of experiencing more severe and frequent ocular symptoms than users of a single tobacco product. Cigarette users experienced more ocular symptoms than e-cigarette users, and those who used these products more recently reported more symptoms than others.

**Meaning:**

The findings of this study provide additional possible reasons for users of e-cigarettes and cigarettes to reduce their tobacco use to prevent or minimize ocular symptoms.

## Teaser

This cross-sectional study examines ocular symptoms among adolescents and young adults using electronic cigarettes compared with cigarettes or both products.

## Introduction

Since 2014, electronic cigarettes (e-cigarettes) have been the most common tobacco product among youth in the US, with multiple tobacco product use becoming increasingly popular.^1,2,3^ E-cigarette use has been linked to numerous adverse outcomes, including increased blood pressure, heart rate, air resistance in lungs, and immunomodulatory cytokines production.^4,5^ Studies examining the dual use of e-cigarettes and cigarettes have reported additional detrimental findings, such as lower general health, increased sleep latency, and higher vascular disorder risk.^6,7,8,9^

Studies on the outcomes of tobacco on the eye have shown that cigarette use is associated with ocular damage and increased eye disease risk, including uveitis, thyroid-associated ophthalmopathy, age-related macular degeneration, glaucoma, and cataract.^10,11,12^ The literature regarding the short- and long-term ocular effects of e-cigarette use is limited.^3,13,14,15,16^ To our knowledge, no study has examined the severity and frequency of ocular symptoms among adolescents and young adults who use e-cigarettes, cigarettes, or both.

This study describes the magnitude of ocular symptoms reported by adolescents and young adults using e-cigarettes only, cigarettes only, and both products (dual users). We hypothesized that dual users would have more frequent and severe ocular symptoms than those who used 1 product, those who used 1 or both products in the past 7 days would have more ocular symptoms than those who used these products in the past 30 days, and those who used 1 or both products in the past 30 days would have more ocular symptoms than those who used these products at least once in their lifetime.

## Methods

### Study Design

From May 6 to May 14, 2020, we conducted an observational cross-sectional survey of e-cigarette use among adolescents and young adults aged 13 to 24 years in the US (N = 4351) via Qualtrics. Qualtrics recruited participants through various strategies (ie, website intercept, gaming sites, customer loyalty portals, and social media). The online sample was balanced for gender and race and ethnicity (as per the latest US census data). It was important to include race and ethnicity in the analyses as studies show that there are racial and ethnic differences in cigarette and e-cigarette use.^17,18,19^ We used quota sampling to purposely balance the proportion of e-cigarette ever users (50.2%) and nonusers (49.8%) in our sample, and equal proportions of participants stratified by 3 age groups (age 13-17 years, termed adolescents [33.7%], age 18-20 years [41.6%] and 21-24 years [24.7%], collectively referred to as young adults in this study).^20^ The institutional review board at Stanford University approved this study. The study adhered to the tenets of the Declaration of Helsinki.^21^ Participants who were interested in contributing to our study expressed their consent and agreement online before commencing the survey. They were informed that they had the possibility to withdraw from the study at any point without any consequences. In addition, participants received monetary compensation for their involvement. This study follows the Strengthening the Reporting of Observational Studies in Epidemiology (STROBE) reporting guideline.

### Measures

Participants were asked sociodemographic questions about their age, gender, and race and ethnicity (eAppendix in Supplement 1).^20^ Participants also were asked whether they had ever used e-cigarette and cigarettes, even just a puff (ever users). Ever users were then asked whether they had used in the past 30 days (past 30-day users). Past 30-day users were then asked whether they had used it in the past 7 days (past 7-day users). Participants answered similar questions regarding their use of other combustible substances, including blunts, cigars/cigarillos, and smoked cannabis (eAppendix in Supplement 1).

We classified the participants based on their reported tobacco use: e-cigarette only users, cigarette only users, and dual users. For each classification, we had a series of pairs: (1) ever users vs never users, (2) past 30-day users vs past 30-day nonusers (among the entire sample, including ever users who did not use in the past 30 days), and (3) past 7-day users vs past 7-day nonusers (among the entire sample, including ever users and past 30-day users who did not use in the past 7 days).

#### General Vision

Participants were asked validated general vision questions. Two questions were from the National Eye Institute Visual Function Questionnaire 25: current eyesight rating (excellent, good, fair, poor, very poor, and completely blind) with glasses or contact lenses if they wear them, and the amount of time spent worrying about their eyesight (none of the time, a little of the time, some of the time, most of the time, and all of the time).^13^ Participants were asked whether they used contact lenses. Those who responded yes answered questions about their contact lens use timing (daytime, nighttime, and during the day and the night) and frequency of use (once per month or less, a few times per month, 1-3 days or nights per week, 4-6 days or nights per week, and every day or night).

#### Frequency and Severity of Ocular Symptoms

Participants indicated frequency (never, 1 time per month or less, 2-3 times per month, 1-2 times per week, 3-4 times per week, 5-6 times per week, 1 time per day, 2-3 times per day, 4-5 times per day, and 6 or more times per day) and severity (none, mild, moderate, severe, very severe, with severe and very severe combined to the single very severe category for analyses) of 10 symptoms occurring in and around their eyes: ocular discomfort, pain/aching, burning/stinging, itching, redness, dryness/gritty sensation, glare/sensitivity to light, blurry vision, tired/eye strain, and headaches.

### Statistical Analysis

We analyzed 9 tobacco use patterns using the entire sample: (1) e-cigarette only ever users vs never users, (2) e-cigarette only past 30-day users vs past 30-day nonusers; (3) e-cigarette only past 7-day users vs past 7-day nonusers, (4) cigarette only ever users vs never users, (5) cigarette only past 30-day users vs past 30-day nonusers, (6) cigarette only past 7-day users vs past 7-day nonusers, (7) dual ever users (those who used both e-cigarettes and cigarettes) vs never users, (8) past 30-day dual users vs past 30-day dual nonusers, and (9) past 7-day dual users vs past 7-day dual nonusers.

We performed weighted multivariable logistic regressions to assess the association between frequency and severity of the 10 ocular symptoms and each of these product use patterns. Each regression model was adjusted for sociodemographic factors (age, gender, and race and ethnicity), contact lens use, and use of other combustible substances (blunts, cigars/cigarillos, and smoked cannabis). Statistics were weighted to derive population estimates. Details about measures, weights, and proportions can be found in the report of Gaiha et al.^20^ Fewer than 5% of the sample was missing values regarding their e-cigarette or cigarette use, and these individuals were excluded from the multivariate models. Calculations were conducted using 95% CIs and adjusted odds ratios (AORs). All *P* values were 2-sided and were not adjusted for multiple analyses. All tests were performed in Stata, version 16.1 (StataCorp LLC).

## Results

### Sample Characteristics

Participants had a mean (SD) age of 19.1 (2.9) years; 63.8% identified as female, 36.2% as male, and 43.0% as White (non-Hispanic). Among the total of 4351 participants, 2183 (50.2%) used e-cigarettes and 1588 (36.5%) smoked cigarettes at least once in their life (ever users). Among the 2183 e-cigarette ever users, 55.9% also used cigarettes (dual users). Among the 1092 past 30-day users, 44.3% were dual users. Among the 919 past 7-day users, 45.8% were dual users. Among all participants, 54.8% used other combustible substances (46.2% smoked cannabis, 40.8% used blunts, and 25.7% used cigars, little cigars, or cigarillos) at least once in their lifetime. Detailed sociodemographic characteristics are reported in Table 1.

**Table 1.** Participant Characteristics by E-Cigarette Use Status

Factor	Weighted %			
	Never users	Ever users	Past 30-d users	Past 7-d users
Sample, No.	2168	2183	1092	919
Demographic characteristics				
Age, mean (SD), y	18.6 (2.9)	19.5 (2.9)	20.0 (2.8)	20.2 (2.8)
Gender				
Male	34.5	34.7	41.7	46.5
Female	63.8	63.7	56.8	52.2
Other^a^	1.7	1.6	1.6	1.3
Race and ethnicity				
African American/Black (non-Hispanic)	20.2	20.1	19.7	19.7
Asian, Native Hawaiian, or Pacific Islander (non-Hispanic)	6.8	8.5	8.0	8.3
Hispanic (regardless of race)	18.3	28.2	29.1	29.0
White (non-Hispanic)	50.5	37.5	36.6	36.0
Other^b^	4.2	5.7	6.6	7.0
General vision-related outcomes				
Eyesight				
Completely blind	0.6	0.4	0.2	0.3
Very poor	2.5	5.3	6.0	7.0
Poor	9.3	10.8	13.5	13.5
Good	44.3	44.6	44.7	44.6
Excellent	43.3	38.9	35.6	34.6
Worried about eyesight				
None of the time	37.8	37.8	36.4	34.2
Little of the time	33.5	31.6	32.4	32.5
Some of the time	17.8	18.2	17.8	18.7
Most of the time	7.0	8.9	9.7	11.0
All of the time	3.8	3.6	3.7	3.5
Contact lens wearers	21.7	30.4	35.5	37.2
Other products inhaled				
Cigarette use	9.6	55.9	44.3	45.8
Other combustible use	22.2	78.2	71.7	70.1
Blunts	14.4	59.8	49.0	43.5
Cigars and variants^c^	7.8	38.6	30.7	33.0
Smoked marijuana	15.9	68.1	57.5	52.8

^a^ Other refers to nonbinary or chose not to specify.

^b^ Other refers to American Indian, Alaska Native, multiracial, not Hispanic, and prefer not to answer.

^c^ Cigars and variants refer to cigars, cigarillos, and little cigars.

Participants rated their eyesight as good (44.5%), followed by excellent (40.8%), poor (10.2%), very poor (4.1%), and completely blind (0.5%). When asked how often they were worried about their eyesight, 37.8% of the participants replied none of the time, 32.4% little of the time, 18.0% some of the time, 8.1% most of the time, and 3.7% all the time. A total of 26.7% of the participants were contact lens wearers. Between 1.1% and 3.9% of ever dual users reported severe to very severe ocular symptoms; between 0.9% and 4.3% reported daily symptoms. Regarding severity of ocular symptoms, 18.6% to 29.3% of the participants reported mild symptoms, 6.7% to 21.2% moderate symptoms, and 3.1% to 9.7% severe to very severe symptoms (eTable 1 in Supplement 1). Regarding frequency of ocular symptoms, 16.6% to 33.8% of the participants reported monthly symptoms (1-3 times per month), 6.5% to 18.6% weekly symptoms (1-6 times per week), and 3.0% to 9.3% daily symptoms (eTable 1 in Supplement 1).

### Severity of Ocular Symptoms Associated With Product Use

After adjusting for sociodemographic factors, contact lens use, and use of other combustible substances (Table 2), participants who used only combustible cigarettes at least once in their life had a higher likelihood of experiencing more severe ocular burning/stinging (AOR, 2.68; 95% CI, 1.56-4.62; *P* < .001) and blurry vision (AOR, 2.70; 95% CI, 1.41-5.17; *P* = .003) compared with all other participants (ie, the rest of the entire sample). Participants who used both e-cigarettes and cigarettes at least once in their life (dual ever users) had a higher likelihood of experiencing more severe ophthalmic dryness/gritty sensation (AOR, 1.60; 95% CI, 1.05-2.43; *P* = .03) and blurry vision (AOR, 1.79; 95% CI, 1.21-2.64; *P* = .003) compared with all other participants.

**Table 2.** Participants’ Ocular Symptoms by Severity Based on E-Cigarette, Cigarette, and Dual Use^a^

Ocular symptoms by severity	Ever users vs never users		Past 30-d users vs sample not using in the past 30 d		Past 7-d users vs sample not using in the past 7 d	
	AOR (95% CI)	*P* value	AOR (95% CI)	*P* value	AOR (95% CI)	*P* value
**Discomfort**						
E-cigarette use only	0.87 (0.61-1.24)	.46	1.00 (0.69-1.45)	.96	1.08 (0.72-1.63)	.69
Cigarette use only	1.51 (0.79-2.90)	.21	1.04 (0.54-1.98)	.89	1.47 (0.80-2.71)	.21
Dual use	1.18 (0.81-1.72)	.37	2.10 (1.33-3.32)	.001	2.19 (1.30-3.70)	.003
**Pain/aching**						
E-cigarette use only	0.83 (0.57-1.22)	.36	0.93 (0.63-1.37)	.72	0.93 (0.61-1.43)	.76
Cigarette use only	1.31 (0.64-2.70)	.45	1.54 (0.80-2.93)	.19	1.75 (0.95-3.20)	.07
Dual use	1.24 (0.83-1.86)	.28	1.99 (1.27-3.13)	.003	2.06 (1.24-3.42)	.005
**Burning/stinging**						
E-cigarette use only	1.20 (0.82-1.75)	.34	0.91 (0.63-1.30)	.61	1.05 (0.71-1.54)	.79
Cigarette use only	2.68 (1.56-4.62)	<.001	0.92 (0.49-1.74)	.82	1.42 (0.67-2.98)	.35
Dual use	1.19 (0.78-1.80)	.42	1.81 (1.09-2.99)	.02	2.08 (1.18-3.66)	.01
**Itching**						
E-cigarette use only	1.08 (0.79-1.49)	.61	0.92 (0.66-1.28)	.63	0.99 (0.67-1.46)	.97
Cigarette use only	1.62 (0.83-3.16)	.15	0.83 (0.46-1.49)	.54	0.75 (0.40-1.37)	.35
Dual use	1.07 (0.74-1.55)	.71	1.83 (1.11-3.02)	.02	2.37 (1.36-4.13)	.002
**Redness**						
E-cigarette use only	1.05 (0.73-1.50)	.78	1.10 (0.75-1.62)	.60	1.42 (0.93-2.16)	.10
Cigarette use only	1.79 (0.79-4.04)	.16	0.75 (0.37-1.53)	.44	0.90 (0.46-1.77)	.78
Dual use	1.37 (0.92-2.06)	.12	1.98 (1.23-3.17)	.004	2.58 (1.50-4.46)	.001
**Dryness/gritty sensation**						
E-cigarette use only	0.91 (0.62-1.33)	.64	1.02 (0.67-1.55)	.91	1.09 (0.67-1.77)	.71
Cigarette use only	1.75 (0.94-3.28)	.08	1.42 (0.78-2.61)	.25	1.21 (0.65-2.25)	.53
Dual use	1.60 (1.05-2.43)	.03	2.65 (1.61-4.36)	<.001	2.89 (1.64-5.08)	<.001
**Glare/light sensitivity**						
E-cigarette use only	0.76 (0.53-1.11)	.16	0.76 (0.53-1.09)	.14	0.98 (0.66-1.46)	.95
Cigarette use only	1.38 (0.66-2.87)	.38	1.01 (0.52-1.96)	.98	1.57 (0.79-3.10)	.19
Dual use	1.15 (0.77-1.72)	.49	2.10 (1.32-3.33)	.002	2.56 (1.50-4.35)	.001
**Blurry vision**						
E-cigarette use only	0.83 (0.58-1.20)	.34	0.81 (0.54-1.19)	.29	1.08 (0.70-1.64)	.72
Cigarette use only	2.70 (1.41-5.17)	.003	2.34 (1.17-4.67)	.02	3.66 (1.67-7.98)	.001
Dual use	1.79 (1.21-2.64)	.003	2.19 (1.32-3.65)	.002	2.47 (1.36-4.50)	.003
**Headaches**						
E-cigarette use only	0.86 (0.63-1.17)	.35	0.97 (0.69-1.36)	.87	1.09 (0.73-1.62)	.65
Cigarette use only	1.24 (0.56-2.78)	.59	1.04 (0.48-2.25)	.90	1.49 (0.73-3.02)	.26
Dual use	1.09 (0.77-1.55)	.61	1.78 (1.15-2.78)	.01	2.31 (1.34-4.00)	.003
**Tired/strain**						
E-cigarette use only	0.77 (0.56-1.06)	.11	0.94 (0.66-1.32)	.73	1.10 (0.74-1.64)	.61
Cigarette use only	1.42 (0.82-2.45)	.21	0.85 (0.44-1.64)	.64	1.44 (0.76-2.74)	.26
Dual use	1.10 (0.76-1.58)	.59	1.55 (0.93-2.60)	.09	2.07 (1.11-3.83)	.02

Abbreviations: AOR, adjusted odds ratio; e-cigarette, electronic cigarette.

^a^ Weighted values. Dual users are participants who use both e-cigarettes and cigarettes. This outcomes table is based on regression models that adjusted for sociodemographic factors including age, gender, race and ethnicity, contact lens use, and use of other combustible tobacco and cannabis products (blunts; cigars, little cigars, and cigarillos; and smoked cannabis). The reference is the group using neither e-cigarettes nor cigarettes. The test value of frequency is the Wald test.

Past 30-day users of only combustible cigarettes had a higher likelihood of having more severe blurry vision (AOR, 2.34; 95% CI, 1.17-4.67; *P* = .02) compared with all other participants. Past 30-day dual users were most likely to have more severe ophthalmic discomfort (AOR, 2.10; 95% CI, 1.33-3.32; *P* = .001), pain/aching (AOR, 1.99; 95% CI, 1.27-3.13; *P* = .003), burning/stinging (AOR, 1.81; 95% CI, 1.09-2.99; *P* = .02), itching (AOR, 1.83; 95% CI, 1.11-3.02; *P* = .02), redness (AOR, 1.98; 95% CI, 1.23-3.17; *P* = .004), dryness/gritty sensation (AOR, 2.65; 95% CI, 1.61-4.36; *P* < .001), glare/light sensitivity (AOR, 2.10; 95% CI, 1.32-3.33; *P* = .002), blurry vision (AOR, 2.19; 95% CI, 1.32-3.65; *P* = .002), and headaches (AOR, 1.78; 95% CI, 1.15-2.78; *P* = .01) compared with all other participants (Table 2).

Past 7-day users of only combustible cigarettes had a higher likelihood of having more severe blurry vision (AOR, 3.66; 95% CI, 1.67-7.98; *P* = .001) compared with all other participants. Past 7-day dual users were most likely to have more severe ophthalmic discomfort (AOR, 2.19; 95% CI, 1.30-3.70; *P* = .003), pain/aching (AOR, 2.06; 95% CI, 1.24-3.42; *P* = .005), burning/stinging (AOR, 2.08; 95% CI, 1.18-3.66; *P* = .01), itching (AOR, 2.37; 95% CI, 1.36-4.13; *P* = .002), redness (AOR, 2.58; 95% CI, 1.50-4.46; *P* = .001), dryness/gritty sensation (AOR, 2.89; 95% CI, 1.64-5.08; *P* < .001), glare/light sensitivity (AOR, 2.56; 95% CI, 1.50-4.35; *P* = .001), blurry vision (AOR, 2.47; 95% CI, 1.36-4.50; *P* = .003), headaches (AOR, 2.31; 95% CI, 1.34-4.00; *P* = .003), and tired/strain (AOR, 2.07; 95% CI, 1.11-3.83; *P* = .02) compared with all other participants (Table 2).

### Frequency of Ocular Symptoms Associated With Product Use

After adjusting for sociodemographic factors, contact lens use, and use of other combustible substances (Table 3), participants who ever used only combustible cigarettes at least once in their life had a higher likelihood of experiencing more frequent ocular burning/stinging (AOR, 2.34; 95% CI, 1.43-3.83; *P* = .001) and dryness/gritty sensation (AOR, 2.15; 95% CI, 1.19-3.87; *P* = .01) compared with all other participants. Dual ever users had a higher likelihood of experiencing frequent ophthalmic pain/aching (AOR, 1.69; 95% CI, 1.13-2.53; *P* = .01) and blurry vision (AOR, 1.63; 95% CI, 1.13-2.36; *P* = .009) compared with all other participants.

**Table 3.** Participants’ Ocular Symptoms by Frequency Based on E-Cigarette, Cigarette, and Dual Use^a^

Ocular symptoms by frequency	Ever users vs never users		Past 30-d users vs sample not using in the past 30 d		Past 7-d users vs sample not using in the past 7 d	
	AOR (95% CI)	*P* value	AOR (95% CI)	*P* value	AOR (95% CI)	*P* value
**Discomfort**						
E-cigarette use only	0.94 (0.66-1.35)	.77	1.03 (0.71-1.51)	.85	1.15 (0.77-1.73)	.47
Cigarette use only	0.97 (0.55-1.70)	.93	0.94 (0.47-1.91)	.88	1.48 (0.67-3.26)	.33
Dual use	1.23 (0.82-1.84)	.31	1.90 (1.19-3.02)	.007	2.21 (1.30-3.74)	.003
**Pain/aching**						
E-cigarette use only	1.00 (0.68-1.47)	.99	1.05 (0.71-1.55)	.80	1.31 (0.87-1.97)	.19
Cigarette use only	1.25 (0.66-2.34)	.48	1.94 (0.95-3.97)	.07	2.93 (1.35-6.37)	.006
Dual use	1.69 (1.13-2.53)	.01	3.33 (2.12-5.21)	<.001	3.45 (2.09-5.68)	<.001
**Burning/stinging**						
E-cigarette use only	0.93 (0.65-1.33)	.71	0.79 (0.55-1.15)	.23	0.97 (0.65-1.45)	.92
Cigarette use only	2.34 (1.43-3.83)	.001	0.97 (0.51-1.85)	.94	1.16 (0.64-2.11)	.61
Dual use	1.22 (0.82-1.82)	.32	2.26 (1.41-3.61)	.001	3.08 (1.86-5.09)	<.001
**Itching**						
E-cigarette use only	1.04 (0.76-1.43)	.79	0.96 (0.67-1.36)	.83	1.11 (0.77-1.62)	.55
Cigarette use only	1.34 (0.68-2.62)	.39	0.82 (0.44-1.54)	.55	1.06 (0.60-1.90)	.82
Dual use	1.01 (0.72-1.43)	.93	1.22 (0.80-1.86)	.34	1.49 (0.94-2.36)	.09
**Redness**						
E-cigarette use only	1.02 (0.71-1.46)	.89	0.97 (0.65-1.43)	.89	1.32 (0.86-2.01)	.20
Cigarette use only	1.62 (0.84-3.15)	.15	0.89 (0.42-1.88)	.78	1.13 (0.57-2.20)	.72
Dual use	1.38 (0.93-2.03)	.10	2.00 (1.29-3.10)	.002	2.72 (1.69-4.36)	<.001
**Dryness/gritty sensation**						
E-cigarette use only	1.14 (0.76-1.69)	.51	0.98 (0.64-1.49)	.94	1.25 (0.80-1.98)	.32
Cigarette use only	2.15 (1.19-3.87)	.01	1.04 (0.55-1.96)	.88	1.29 (0.70-2.38)	.41
Dual use	1.50 (0.98-2.29)	.06	1.89 (1.17-3.07)	.009	2.16 (1.26-3.70)	.005
**Glare/light sensitivity**						
E-cigarette use only	0.85 (0.60-1.21)	.38	0.96 (0.67-1.39)	.85	1.29 (0.86-1.93)	.21
Cigarette use only	0.97 (0.57-1.65)	.92	1.29 (0.72-2.30)	.39	1.54 (0.89-2.66)	.12
Dual use	1.12 (0.78-1.62)	.53	1.73 (1.08-2.76)	.02	2.26 (1.31-3.90)	.003
**Blurry vision**						
E-cigarette use only	0.83 (0.59-1.17)	.31	0.76 (0.53-1.08)	.14	0.94 (0.64-1.38)	.78
Cigarette use only	1.56 (0.88-2.76)	.13	2.43 (1.27-4.64)	.007	3.15 (1.61-6.15)	.001
Dual use	1.63 (1.13-2.36)	.009	1.76 (1.13-2.75)	.01	2.20 (1.27-3.81)	.005
**Headaches**						
E-cigarette use only	0.79 (0.59-1.05)	.12	0.96 (0.70-1.30)	.81	1.03 (0.72-1.48)	.84
Cigarette use only	1.04 (0.56-1.94)	.88	0.96 (0.45-2.06)	.93	1.84 (0.76-4.43)	.17
Dual use	0.92 (0.65-1.31)	.68	1.97 (1.22-3.18)	.005	2.15 (1.26-3.68)	.005
**Tired/strain**						
E-cigarette use only	0.82 (0.59-1.13)	.24	0.88 (0.61-1.25)	.49	1.11 (0.78-1.58)	.55
Cigarette use only	1.23 (0.73-2.07)	.42	1.15 (0.60-2.21)	.67	1.74 (0.81-3.74)	.15
Dual use	1.09 (0.76-1.56)	.61	1.59 (0.96-2.64)	.07	1.95 (1.06-3.58)	.03

Abbreviations: AOR, adjusted odds ratio; e-cigarette, electronic cigarette.

^a^ Weighted values. Dual users are participants who use both e-cigarettes and cigarettes. This outcomes table is based on regression models that adjusted for sociodemographic factors including age, gender, race and ethnicity, contact lens use, and use of other combustible tobacco and cannabis products (blunts; cigars, little cigars, and cigarillos; and smoked cannabis). The reference is the group using neither e-cigarettes nor cigarettes. The test value of frequency is the Wald test.

Past 30-day users of only combustible cigarettes had a higher likelihood of having more frequent blurry vision (AOR, 2.43; 95% CI, 1.27-4.64; *P* = .007) compared with all other participants. Past 30-day dual users were more likely to have frequent ophthalmic discomfort (AOR, 1.90; 95% CI, 1.19-3.02; *P* = .007), pain/aching (AOR, 3.33; 95% CI, 2.12-5.21; *P* < .001), burning/stinging (AOR, 2.26; 95% CI, 1.41-3.61; *P* = .001), redness (AOR, 2.00; 95% CI, 1.29-3.10; *P* = .002), dryness/gritty sensation (AOR, 1.89; 95% CI, 1.17-3.07; *P* = .009), glare/light sensitivity (AOR, 1.73; 95% CI, 1.08-2.76; *P* = .02), blurry vision (AOR, 1.76; 95% CI, 1.13-2.75; *P* = .01), and headaches (AOR, 1.97; 95% CI, 1.22-3.18; *P* = .005) compared with all other participants (Table 3).

Past 7-day users of combustible cigarettes only had a higher likelihood of having more frequent pain/aching (AOR, 2.93; 95% CI, 1.35-6.37; *P* = .006), and blurry vision (AOR, 3.15; 95% CI, 1.61-6.15; *P* = .001) compared with all other participants. Past 7-day dual users were more likely to have frequent ophthalmic discomfort (AOR, 2.21; 95% CI, 1.30-3.74; *P* = .003), pain/aching (AOR, 3.45; 95% CI, 2.09-5.68; *P* < .001), burning/stinging (AOR, 3.08; 95% CI, 1.86-5.09; *P* < .001), redness (AOR, 2.72; 95% CI, 1.69-4.36; *P* < .001), dryness/gritty sensation (AOR, 2.16; 95% CI, 1.26-3.70; *P* = .005), glare/light sensitivity (AOR, 2.26; 95% CI, 1.31-3.90; *P* = .003), blurry vision (AOR, 2.20; 95% CI, 1.27-3.81; *P* = .005), headaches (AOR, 2.15; 95% CI, 1.26-3.68; *P* = .005), and tired/strain (AOR, 1.95; 95% CI, 1.06-3.58; *P* = .03) compared with all other participants (Table 3).

### Past 7 and 30 Days Dual Users

Participants who used both cigarettes and e-cigarettes with a lower frequency (past 30-day users) were consistently more likely to develop ocular symptoms compared with those who did not use either. Dual users who used cigarettes with higher frequency (past 7-day use) and e-cigarettes with lower frequency (past 30-day use), as well as dual users who used e-cigarettes with higher frequency (past 7-day use) and cigarettes with lower frequency (past 30-day use) did not differ significantly in their risk of developing ocular symptoms (eTable 2, eTable 3, and eTable 4 in Supplement 1).

### General Vision-Related Outcomes

Dual ever users (AOR, 0.64; 95% CI, 0.46-0.90; *P* = .01) and ever users of cigarettes only (AOR, 0.51; 95% CI, 0.34-0.76; *P* = .001) were more likely to report poorer eyesight. Cigarette only users were more likely to report poorer eyesight compared with all other participants in the past 30 days (AOR, 0.48; 95% CI, 0.29-0.81; *P* = .006) and the past 7 days (AOR, 0.49; 95% CI, 0.30-0.79; *P* = .004). After adjusting for sociodemographic factors, contact lens use, and use of other combustible substances, e-cigarette only ever users (AOR, 0.60; 95% CI, 0.44-0.82; *P* = .002), past 30-day e-cigarette only users (AOR, 0.54; 95% CI, 0.38-0.78; *P* = .001), and past 7-day e-cigarette only users (AOR, 0.64; 95% CI, 0.44-0.93; *P* = .02) were more likely to be worried about their eyesight compared with all other participants (Table 4).

**Table 4.** Participants’ General Vision-Related Outcomes Based on E-Cigarette, Cigarette, and Dual Use^a^

General vision-related outcomes	Ever users vs never users		Past 30-d users vs sample not using in the past 30 d		Past 7-d users vs sample not using in the past 7 d	
	AOR (95% CI)	*P* value	AOR (95% CI)	*P* value	Odds ratio (95% CI)	*P* value
**Eyesight**						
E-cigarette use only	1.12 (0.82-1.52)	.47	0.88 (0.62-1.25)	.49	0.84 (0.57-1.25)	.41
Cigarette use only	0.51 (0.34-0.76)	.001	0.48 (0.29-0.81)	.006	0.49 (0.30-0.79)	.004
Dual use	0.64 (0.46-0.90)	.01	0.72 (0.45-1.16)	.18	0.68 (0.37-1.28)	.24
**Worried about eyesight**						
E-cigarette use only	0.60 (0.44-0.82)	.002	0.54 (0.38-0.78)	.001	0.64 (0.44-0.93)	.02
Cigarette use only	1.18 (0.81-1.71)	.38	1.27 (0.76-2.11)	.34	1.07 (0.70-1.62)	.75
Dual use	1.14 (0.82-1.60)	.42	1.28 (0.85-1.93)	.23	1.20 (0.72-2.01)	.48

Abbreviations: AOR, adjusted odds ratio; e-cigarette, electronic cigarette.

^a^ Weighted values. Dual users are participants who use both e-cigarettes and cigarettes. This outcomes table is based on regression models that adjusted for sociodemographic factors including age, gender, race and ethnicity, contact lens use, and use of other combustible tobacco and cannabis products (blunts; cigars, little cigars, and cigarillos; and smoked cannabis). The reference is the group using neither e-cigarettes nor cigarettes. The test value of frequency is the Wald test.

## Discussion

This study examined US adolescents’ and young adults’ reported frequency and severity of a comprehensive list of ocular symptoms by e-cigarette, cigarette, and dual use. While previous work in this area has studied occupational exposure or incidental unintentional injuries related to e-cigarette misuse or dysfunction,^3,14,15,16,22,23,24,25,26^ this study focused on a target population with a high prevalence of e-cigarette use.^3,14,15^ In our study, dual use of e-cigarettes and cigarettes was associated with a higher severity and frequency of ocular symptoms among adolescents and young adults in the US. Past 7-day users experienced more symptoms than past 30-day users, and past 30-day users experienced more symptoms than ever users. Cigarette-only users also experienced ocular symptoms, but to a lesser degree than dual users. Despite no association with e-cigarette use alone for any outcome, e-cigarette only users were most worried about their eyesight. Future studies should ascertain the reasons for differences in concern over eyesight among different tobacco users.

Irrespective of the pattern of tobacco product use, most participants reported having no symptoms or mild ocular symptoms, occurring up to 3 times per month. Dual users reported moderate to very severe ocular symptoms, which is more severe when compared with e-cigarette only or cigarette only users across usage patterns. Similarly, dual users were found to have more frequent ocular symptoms occurring daily or 1 to 6 times per week compared with e-cigarette only or cigarette only users in each user timeframe. When examining past 30-day and past 7-day dual users, low-frequency dual users were more likely to develop significant ocular symptoms. This supports the overall findings that dual users were at increased risk of ocular symptoms, regardless of the intensity (frequency) of cigarette and e-cigarette use.

The literature on the short-term and long-term outcomes of e-cigarette use as well as dual use of e-cigarettes and cigarettes is scarce.^16,22,23^ Some studies, mainly case reports, have described ocular injuries from vaping-related explosions,^24,25^ vape-pen defects,^26^ and e-liquid misuse.^27,28,29,30^ One case study discussed the development of eye cancer in a young e-cigarette user,^31^ and another study assessed workers’ chemical exposures in vape shops.^32^ Experimental studies have examined the outcomes of vaping associated with anterior corneal surface integrity,^33^ tear function,^34^ and retinal tissue development.^35,36^ One cross-sectional US-based population study reported an association between e-cigarette use and visual impairment in individuals older than 18 years.^37^ Published between 2019 and 2022, these studies have since largely been the basis of the gray literature associating vaping and eyesight. The emphasis on the potential harmful visual and general health effects of e-cigarettes in the gray literature might be one of the reasons why e-cigarette use was associated with increased concerns regarding eyesight. In our study, e-cigarette users were found to report less severe and frequent ocular symptoms than cigarette users.

These findings may be attributable to several reasons. Smoking may be a risk factor for dry eye disease.^38^ As combustible cigarette puffs involve free radicals, cigarette use can increase oxidative stress and inflammation. The oxidative attacks produced by radicals can damage the tear film lipid and may lead to ocular symptoms seen in dry eye disease.^31^ In a pilot study of male e-cigarette users and healthy nonsmokers, e-cigarette users experienced moderate to severe dry eye as per the ocular surface disease index, considering light sensitivity, grittiness feeling, painful or sore eyes, blurred vision, and poor vision.^28^ In these e-cigarette users, the noninvasive tear breakup time was reduced, meaning that they had decreased tear film quality, which can lead to more severe and frequent ocular symptoms. The single-visit study further hypothesized that the solvent for e-cigarette liquid (propylene glycol) generates free radicals destroying the lipid layer of tear film via lipid peroxidation. Therefore, it is plausible that both cigarettes and e-cigarettes induce oxidative stress and can be implicated in processes causing vasoconstriction in the eye.^29,32,33,34^ Given the limited number of studies and changing landscape of e-cigarette products, the conclusion on e-cigarette use is not unanimous: a study on the acute effects of e-cigarette use reported that mild exposure to e-cigarettes does not significantly affect the corneal epithelial thickness and noninvasive keratography tear break up.^27^

### Limitations

This study has limitations. Causal or temporal association between ocular symptoms and e-cigarette and cigarette use could not be determined given the observational cross-sectional study design.^39,40,41^ The findings cannot be generalized to the adolescent and young adult population outside of the US, given that our sample was restricted to US residents.^42,43,44,45^ While we controlled for potential confounders (age, gender, race and ethnicity, and use of other combustible substances), there might be other confounders (eg, screen time, facemask use during the COVID-19 pandemic) that can lead to increased ocular symptoms. Additionally, some reported outcomes may be due to chance as multiple statistical tests were performed. There is also possibility of self-reported bias.^46,47^ Self-reported responses can be understated or exaggerated by participants, and participants might not recall exactly how often they use e-cigarettes and cigarettes and the frequency and severity of the ocular symptoms.^48,49,50^ Severity is also a notion that is not objectively standardized or quantified for participants.^51,52^

Consistent with the standard method used in the field of tobacco research, we categorized participants as never users, ever users, past 30 days users, and past 7 days users.^53^ Given that cigarette dosage (number of cigarettes) cannot be directly translated or applied to e-cigarette dosage, we were unable to compare both products in terms of mild, moderate, and heavy use. Additionally, it is difficult to conclude that dual users were showing poorer outcomes solely due to an interaction between use of 2 different tobacco products given the small sample sizes (eTable 2 in the Supplement).

## Conclusions

This cross-sectional study found an association between dual use of e-cigarettes and cigarettes and more severe and frequent ocular symptoms among adolescents and young adults in the US. Further longitudinal studies are necessary to validate our findings. These findings provide additional reasons to screen, counsel, and treat all tobacco users to prevent and reduce ocular symptoms. We recommend that health care clinicians ask all patients about nicotinic product use and counsel and treat those using these products to help prevent and reduce ophthalmologic issues.^54^

## Notes

Supplement 1. **eAppendix.** Survey Questionnaire **eTable 1.** Participants’ Ocular Symptoms by Severity and Frequency **eTable 2.** Frequency of Participants Based on Dual Use of e-Cigarette and Cigarette in the Past 7 and the Past 30 Days **eTable 3.** Dual Users’ Ocular Symptoms by Severity Based on Frequency of e-Cigarette and Cigarette Use in the Past 7 and the Past 30 Days **eTable 4.** Dual Users’ Ocular Symptoms by Frequency Based on Frequency of e-Cigarette and Cigarette Use in the Past 7 and the Past 30 Days

Supplement 2.
Data Sharing Statement
