# Supplementary material for: Ocular Symptoms in Adolescents and Young Adults With Electronic Cigarette, Cigarette, and Dual Use
Source: JAMA Ophthalmol. 2023 Aug 31;141(10):937–46. doi: 10.1001/jamaophthalmol.2023.3852 (PMC10472265; doi:10.1001/jamaophthalmol.2023.3852)
Supplement: Supplement 1. — eAppendix. Survey Questionnaire eTable 1. Participants’ Ocular Symptoms by Severity and Frequency eTable 2. Frequency of Participants Based on Dual Use of e-Cigarette and Cigarette in the Past 7 and the Past 30 Days eTable 3. Dual Users’ Ocular Symptoms by Severity Based on Frequency of e-Cigarette and Cigarette Use in the Past 7 and the Past 30 Days eTable 4. Dual Users’ Ocular Symptoms by Frequency Based on Frequency of e-Cigarette and Cigarette Use in the Past 7 and the Past 30 Days [file jamaophthalmol-e233852-s001.pdf]

Supplementary Online Content

Nguyen AX, Gaiha SM, Chung S, Halpern-Felsher B, Wu AY. Ocular symptoms among adolescents and younger adults with electronic cigarette, cigarette, and dual use. *JAMA Ophthalmol*. Published online August 31, 2023. doi:10.1001/jamaophthalmol.2023.3852

eAppendix. Survey Questionnaire

eTable 1. Participants' Ocular Symptoms by Severity and Frequency

eTable 2. Frequency of Participants Based on Dual Use of e-Cigarette and Cigarette in the Past 7 and the Past 30 Days

eTable 3. Dual Users' Ocular Symptoms by Severity Based on Frequency of e-Cigarette and Cigarette Use in the Past 7 and the Past 30 Days

eTable 4. Dual Users' Ocular Symptoms by Frequency Based on Frequency of e-Cigarette and Cigarette Use in the Past 7 and the Past 30 Days

This supplementary material has been provided by the authors to give readers additional information about their work.

© 2023 Nguyen AX et al. *JAMA Ophthalmology*.

eAppendix. Survey Questionnaire

SCREENING QUESTIONS

1. How old are you today?
   (drop down menu from 13-24 years)

2. Have you ever used an e-cigarette or vape before today?
   - o Yes
   - o No

DEMOGRAPHIC INFORMATION

1. Do you identify as…
   - o Female
   - o Male
   - o Non-Binary/ Other
   - o Choose to not specify/indicate

2. What is your ethnicity?
   - o Hispanic or Latino
   - o Not Hispanic or Latino

3. What is your race? Choose one or more if applicable.
   - ☐ American Indian or Alaska Native
   - ☐ Asian
   - ☐ Black or African American
   - ☐ Native Hawaiian or Other Pacific Islander
   - ☐ White
   - ☐ More than one race
   - ☐ Prefer not to answer

PATTERNS OF USE

1. Have you EVER USED any of these products in your entire life? (dropdown with yes/no)

Product	Yes	No
Cigarette, even 1 or 2 puffs		
Cigars, cigarillos or little cigars, even 1 or 2 puffs		
Hookah, even 1 or 2 puffs		
Disposable pod-based vape like Puffbar or FOGG, even 1 or 2 puffs		
Pod-based vape like Juul or Suorin, even 1 or 2 puffs		
Any other vape like mods, even 1 or 2 puffs		
Vaped THC (wax or oil) or marijuana, even 1 or 2 puffs		
Alcohol, even a few sips		
Blunts, even 1 or 2 puffs		
Smoked marijuana, even 1 or 2 puffs		
Edible marijuana, even 1 or 2 bites		

© 2023 Nguyen AX et al. *JAMA Ophthalmology*.

If YES to anything to the question above, only the list that participants identified as ever used will re-appear in any subsequent questions.

2. **HOW MANY TIMES IN YOUR ENTIRE** LIFE have you used these products? (dropdown)

	Never	1-2 times	3-10 times	11-19 times	20-30 times	31-99 times	100 or more times
Only the list that participants identified as ever used							

3. During the **LAST 30 DAYS**, ON ABOUT HOW MANY DAYS did you… (Put a 0 if you did not use the product in the last 30 days)

Product	Number of days (Between 0-30 days)
Only the list that participants identified as ever used	

4. During the **LAST 7 DAYS**, ON ABOUT HOW MANY DAYS did you… (Put a 0 if you did not use the product in the last 30 days)

Product	Number of days (Between 0-7 days)
Only the list that participants identified as ever used	

GENERAL VISION

1. At the present time, how is your **eyesight** using both eyes (with glasses or contact lenses, if you wear them)?

- Excellent
- Good
- Fair
- Poor
- Very poor
- I am completely Blind

2. How much of the time do you worry about your **eyesight**?

- None of the time
- A little of the time
- Some of the time
- Most of the time
- All of the time

3. Do you use **contact lenses**?

- Yes
- No

© 2023 Nguyen AX et al. *JAMA Ophthalmology*.

OCULAR SYMPTOMS

1. At what **frequency** do you experience the following symptoms **in and around your eyes**? (dropdown)

	Never	1 time per month or less	2-3 times per month	1-2 times per week	3-4 times per week	5-6 times per week	1 time per day	2-3 times per day	4-5 times per day	6 or more times per day
Discomfort	o	o	o	o	o	o	o	o	o	o
Pain or aching	o	o	o	o	o	o	o	o	o	o
Burning or stinging	o	o	o	o	o	o	o	o	o	o
Itching	o	o	o	o	o	o	o	o	o	o
Redness	o	o	o	o	o	o	o	o	o	o
Dryness or a gritty sensation	o	o	o	o	o	o	o	o	o	o
Glare or sensitivity to light	o	o	o	o	o	o	o	o	o	o
Blurry vision	o	o	o	o	o	o	o	o	o	o
Headaches	o	o	o	o	o	o	o	o	o	o
Tired or strain	o	o	o	o	o	o	o	o	o	o

© 2023 Nguyen AX et al. *JAMA Ophthalmology*.

2. How would you quantify the symptom's severity? **How much** discomfort, pain, burning, itching, redness, dryness, glare, blurry vision and/or headaches have you had in and around your eyes? (dropdown)

	None	Mild	Moderate	Severe	Very severe
Discomfort	o	o	o	o	o
Pain or aching	o	o	o	o	o
Burning or stinging	o	o	o	o	o
Itching	o	o	o	o	o
Redness	o	o	o	o	o
Dryness or a gritty sensation	o	o	o	o	o
Glare or sensitivity to light	o	o	o	o	o
Blurry vision	o	o	o	o	o
Headaches	o	o	o	o	o
Tired or strain	o	o	o	o	o

© 2023 Nguyen AX et al. *JAMA Ophthalmology*.

eTable 1. Participants' ocular symptoms by severity and frequency (weighted percentages, %).

Ocular symptoms by severity	All users (n=4,351)	Ocular symptoms by frequency	All users (n=4,351)
Discomfort		Discomfort	
None	60.98	Never	59.8
Mild	28.11	1-3 times/month	25.59
Moderate	7.36	1-6 times/week	11.07
(Very) Severe	3.55	At least once a day	3.53
Pain or aching		Pain or aching	
None	68.38	Never	68.81
Mild	21.83	1-3 times/month	21.05
Moderate	6.68	1-6 times/week	6.48
(Very) Severe	3.11	At least once a day	3.66
Burning or stinging		Burning or stinging	
None	67.11	Never	63.87
Mild	19.91	1-3 times/month	22.69
Moderate	8.85	1-6 times/week	10.43
(Very) Severe	4.14	At least once a day	3.02
Itching		Itching	
None	52.38	Never	47.52
Mild	29.29	1-3 times/month	32.41
Moderate	11.95	1-6 times/week	15.20
(Very) Severe	6.38	At least once a day	4.87
Redness		Redness	
None	66.81	Never	63.01
Mild	20.82	1-3 times/month	23.54
Moderate	6.99	1-6 times/week	9.49
(Very) Severe	5.38	At least once a day	3.95
Dryness or gritty sensation		Dryness or gritty sensation	
None	70.66	Never	69.59
Mild	16.54	1-3 times/month	16.64
Moderate	8.25	1-6 times/week	9.16
(Very) Severe	4.55	At least once a day	4.60
Glare or light sensitivity		Glare or light sensitivity	
None	61.68	Never	58.73
Mild	18.71	1-3 times/month	21.86
Moderate	13.88	1-6 times/week	13.03
(Very) Severe	5.74	At least once a day	6.38
Blurry vision		Blurry vision	
None	63.24	Never	57.51
Mild	18.55	1-3 times/month	22.25
Moderate	10.26	1-6 times/week	11.92
(Very) Severe	7.95	At least once a day	8.32

© 2023 Nguyen AX et al. *JAMA Ophthalmology*.

Headaches		Headaches	
None	47.20	Never	42.49
Mild	21.97	1-3 times/month	33.83
Moderate	21.15	1-6 times/week	17.28
(Very) Severe	9.67	At least once a day	6.40
Tired or strain		Tired or strain	
None	51.61	Never	46.25
Mild	24.59	1-3 times/month	25.86
Moderate	16.13	1-6 times/week	18.61
(Very) Severe	7.67	At least once a day	9.28

© 2023 Nguyen AX et al. *JAMA Ophthalmology*.

eTable 2. Frequency of participants based on dual use of e-cigarette and cigarette in the past 7 and the past 30 days.

Ocular symptom	Sample (N)
E-cigarette used in past 7 days and cigarette used in past 7 days	3868
E-cigarette used in past 30 days and cigarette used in past 7 days	4
E-cigarette used in past 7 days and cigarette used in past 30 days	15
E-cigarette used in past 30 days and cigarette used in past 30 days	11
Other[a]	453
Total	4351

[a]Other refers to: e-cigarette never used and cigarette never used; e-cigarette used in past 30 days and cigarette not used in past 30 days; e-cigarette not used in the past 30 days and cigarettes used in past 30 days

© 2023 Nguyen AX et al. *JAMA Ophthalmology*.

eTable 3. Dual users' ocular symptoms by severity based on frequency of e-cigarette and cigarette use in the past 7 and the past 30 days (weighted values).
Note: Dual users are participants who use both e-cigarettes and cigarettes. This outcomes table is based on regression models, which adjust for bio-demographic factors including the following: age, gender, contact lens use, race/ethnicity, and use of other combustible tobacco and cannabis (blunts; cigars, little cigars, and cigarillos; and smoked cannabis). The reference is the "Other" group defined in Supplemental Table 2. The test value of frequency is the Wald test.

Ocular symptom by severity	Odds Ratio (95%CI)	P-value
Discomfort		
E-cigarette used in past 7 days and cigarette used in past 7 days	11.81 (6.45-21.61)	<.001
E-cigarette used in past 30 days and cigarette used in past 7 days	2.05 (0.35-12.01)	.43
E-cigarette used in past 7 days and cigarette used in past 30 days	0.81 (0.13-5.17)	.82
E-cigarette used in past 30 days and cigarette used in past 30 days	2.20 (1.43-3.39)	<.001
Pain / aching		
E-cigarette used in past 7 days and cigarette used in past 7 days	28.98 (0.63-1339.42)	.09
E-cigarette used in past 30 days and cigarette used in past 7 days	3.46 (0.66-18.12)	.14
E-cigarette used in past 7 days and cigarette used in past 30 days	1.11 (0.16-7.80)	.92
E-cigarette used in past 30 days and cigarette used in past 30 days	2.35 (1.53-3.60)	<.001
Burning / stinging		
E-cigarette used in past 7 days and cigarette used in past 7 days	7.86 (2.27-27.22)	.001
E-cigarette used in past 30 days and cigarette used in past 7 days	3.44 (1.43-8.28)	.006
E-cigarette used in past 7 days and cigarette used in past 30 days	0.37 (0.07-2.07)	.26
E-cigarette used in past 30 days and cigarette used in past 30 days	2.05 (1.25-3.37)	.004
Itching		
E-cigarette used in past 7 days and cigarette used in past 7 days	1.46 (0.76-2.80)	.25
E-cigarette used in past 30 days and cigarette used in past 7 days	2.29 (0.67-7.78)	.19
E-cigarette used in past 7 days and cigarette used in past 30 days	0.42 (0.07-2.71)	.36
E-cigarette used in past 30 days and cigarette used in past 30 days	2.30 (1.41-3.76)	.001
Redness		
E-cigarette used in past 7 days and cigarette used in past 7 days	28.74 (1.21-680.25)	.04
E-cigarette used in past 30 days and cigarette used in past 7 days	0.93 (0.16-5.42)	.93
E-cigarette used in past 7 days and cigarette used in past 30 days	1.32 (0.11-15.58)	.83
E-cigarette used in past 30 days and cigarette used in past 30 days	2.47 (1.56-3.90)	<.001
Dryness / gritty sensation		
E-cigarette used in past 7 days and cigarette used in past 7 days	0.18 (0.02-1.82)	.15
E-cigarette used in past 30 days and cigarette used in past 7 days	1.97 (0.22-17.32)	.54
E-cigarette used in past 7 days and cigarette used in past 30 days	1.56 (0.16-15.40)	.71
E-cigarette used in past 30 days and cigarette used in past 30 days	2.65 (1.66-4.21)	<.001
Glare / light sensitivity		
E-cigarette used in past 7 days and cigarette used in past 7 days	2.07 (0.52-8.24)	.30
E-cigarette used in past 30 days and cigarette used in past 7 days	1.07 (0.12-9.31)	.95
E-cigarette used in past 7 days and cigarette used in past 30 days	1.20 (0.18-8.17)	.86
E-cigarette used in past 30 days and cigarette used in past 30 days	2.53 (1.65-3.88)	<.001
Blurry vision		
E-cigarette used in past 7 days and cigarette used in past 7 days	16.43 (0.39-701.18)	.14
E-cigarette used in past 30 days and cigarette used in past 7 days	2.56 (1.07-6.13)	.03
E-cigarette used in past 7 days and cigarette used in past 30 days	1.31 (0.27-6.36)	.73
E-cigarette used in past 30 days and cigarette used in past 30 days	2.59 (1.59-4.21)	<.001

© 2023 Nguyen AX et al. *JAMA Ophthalmology*.

eTable 4. Dual users' ocular symptoms by frequency based on frequency of e-cigarette and cigarette use in the past 7 and the past 30 days (weighted values).
Note: Dual users are participants who use both e-cigarettes and cigarettes. This outcomes table is based on regression models, which adjust for bio-demographic factors including the following: age, gender, contact lens use, race/ethnicity, and use of other combustible tobacco and cannabis (blunts; cigars, little cigars, and cigarillos; and smoked cannabis). The reference is the "Other" group defined in Supplemental Table 2. The test value of frequency is the Wald test.

Ocular symptom by frequency	Odds Ratio (95%CI)	P-value
Discomfort		
E-cigarette used in past 7 days and cigarette used in past 7 days	2.48 (1.48-4.13)	.001
E-cigarette used in past 30 days and cigarette used in past 7 days	2.66 (0.55-12.87)	.22
E-cigarette used in past 7 days and cigarette used in past 30 days	1.07 (0.09-12.26)	.96
E-cigarette used in past 30 days and cigarette used in past 30 days	1.98 (1.29-3.05)	.002
Pain / aching		
E-cigarette used in past 7 days and cigarette used in past 7 days	8.07 (1.64-39.78)	.01
E-cigarette used in past 30 days and cigarette used in past 7 days	2.64 (0.29-23.83)	.39
E-cigarette used in past 7 days and cigarette used in past 30 days	1.07 (0.09-13.19)	.96
E-cigarette used in past 30 days and cigarette used in past 30 days	3.55 (2.32-5.42)	<.001
Burning / stinging		
E-cigarette used in past 7 days and cigarette used in past 7 days	7.76 (1.87-32.23)	.01
E-cigarette used in past 30 days and cigarette used in past 7 days	6.40 (3.70-11.08)	<.001
E-cigarette used in past 7 days and cigarette used in past 30 days	0.72 (0.14-3.79)	.70
E-cigarette used in past 30 days and cigarette used in past 30 days	2.79 (1.78-4.38)	<.001
Redness		
E-cigarette used in past 7 days and cigarette used in past 7 days	7.70 (1.98-29.90)	.003
E-cigarette used in past 30 days and cigarette used in past 7 days	2.20 (0.42-11.47)	.35
E-cigarette used in past 7 days and cigarette used in past 30 days	1.00 (0.07-14.10)	1.00
E-cigarette used in past 30 days and cigarette used in past 30 days	2.55 (1.71-3.78)	<.001
Dryness / gritty sensation		
E-cigarette used in past 7 days and cigarette used in past 7 days	25.66 (0.64-1022.97)	.08
E-cigarette used in past 30 days and cigarette used in past 7 days	2.10 (0.47-9.32)	.33
E-cigarette used in past 7 days and cigarette used in past 30 days	0.40 (0.08-2.09)	.28
E-cigarette used in past 30 days and cigarette used in past 30 days	2.08 (1.37-3.17)	.001
Glare / light sensitivity		
E-cigarette used in past 7 days and cigarette used in past 7 days	5.27 (0.65-43.00)	.12
E-cigarette used in past 30 days and cigarette used in past 7 days	2.55 (0.32-20.50)	.38
E-cigarette used in past 7 days and cigarette used in past 30 days	3.52 (1.78-6.94)	<.001
E-cigarette used in past 30 days and cigarette used in past 30 days	2.11 (1.41-3.15)	<.001
Blurry vision		
E-cigarette used in past 7 days and cigarette used in past 7 days	4.42 (0.68-28.56)	.12
E-cigarette used in past 30 days and cigarette used in past 7 days	38.52 (9.81-151.18)	<.001
E-cigarette used in past 7 days and cigarette used in past 30 days	3.60 (2.08-6.23)	<.001
E-cigarette used in past 30 days and cigarette used in past 30 days	2.26 (1.50-3.41)	<.001
Headaches		
E-cigarette used in past 7 days and cigarette used in past 7 days	17.90 (0.19-1698.89)	.21
E-cigarette used in past 30 days and cigarette used in past 7 days	2.35 (0.13-43.54)	.57
E-cigarette used in past 7 days and cigarette used in past 30 days	0.65 (0.08-5.25)	.69
E-cigarette used in past 30 days and cigarette used in past 30 days	2.02 (1.27-3.23)	.003

© 2023 Nguyen AX et al. *JAMA Ophthalmology*.
