# Supplementary material for: Ocular Symptoms in Adolescents and Young Adults With Electronic Cigarette, Cigarette, and Dual Use
Source: JAMA Ophthalmol. 2023 Aug 31;141(10):937–46. doi: 10.1001/jamaophthalmol.2023.3852 (PMC10472265; doi:10.1001/jamaophthalmol.2023.3852)
Supplement: Supplement 2. — Data Sharing Statement [file jamaophthalmol-e233852-s002.pdf]

Data Sharing Statement

Nguyen. Ocular Symptoms in Adolescents and Young Adults With Electronic Cigarette, Cigarette, and Dual Use. *JAMA Ophthalmol*. Published August 31, 2023. doi:10.1001/jamaophthalmol.2023.3852

Data
Data available: No

Additional Information
Explanation for why data not available: Data will be shared on a case-by-case basis and upon reasonable request. Shared data will need to comply with the regulations set by the Taube Research Faculty Scholar Endowment, the grant U54 HL147127 from the National Heart, Lung, and Blood Institute (NHLBI) and the Food and Drug Administration Center for Tobacco Products, as they supported the research reported and co-led by Bonnie Halpern-Felsher.
